# Acute basophilic leukemia presenting with maculopapular rashes and a gastric ulcer: A case report

**DOI:** 10.3892/ol.2014.2544

**Published:** 2014-09-17

**Authors:** XIAO-HUA LUO, YAN ZHU, XIAO-QIONG TANG

**Affiliations:** 1 Department of Hematology, The First Affiliated Hospital of Chongqing Medical University, Chongqing 400016, P.R. China; 2 Department of Hematology, Southwest Hospital, Third Military Medical University, Chongqing 400038, P.R. China

**Keywords:** acute basophilic leukemia, maculopapular rashes, gastric ulcer

## Abstract

Acute basophilic leukemia (ABL) is a rare and poorly characterized form of leukemia. The case of a 65-year-old male who complained of dizziness, maculopapular skin lesions and melena is described in the current report. A gastroscopy was conducted and indicated a gastric antral ulcer. The diagnosis of ABL was determined due to characteristic cytomorphological features, the myeloid immunophenotype of the blast cells (identified to be positive for cluster of differentiation [CD]25 and CD123) in addition to the absence of the Philadelphia chromosome and a c-kit D816V mutation. The patient initially demonstrated clinical improvement as a result of chemotherapy, however, subsequently deteriorated. The gastric and skin manifestations of ABL may be associated with excessive histamine release from basophilic cells. Thus, the administration of H1- and H2-receptor antagonists, proton pump inhibitors and steroids is proposed in order to minimize these associated complications.

## Introduction

Acute basophilic leukemia (ABL) is a rare hematologic malignancy that is currently poorly described. The clinical progression of ABL is rapid and associated with a poor prognosis. Although ABL is currently recognized as a distinct clinical entity in the classification of myeloid malignancies (1), its rarity complicates the diagnosis of the disease and the establishment of a treatment protocol. In the present report, the case of an elderly male who presented with ABL, as well as a gastric antral ulcer and maculopapular rashes is described. This study was approved by the Ethics Committee of the First Affiliated Hospital of Chongqing Medical University and was performed according to the Declaration of Helsinki. Written informed consent was obtained from the patient’s family.

## Case report

**Figure 1:**
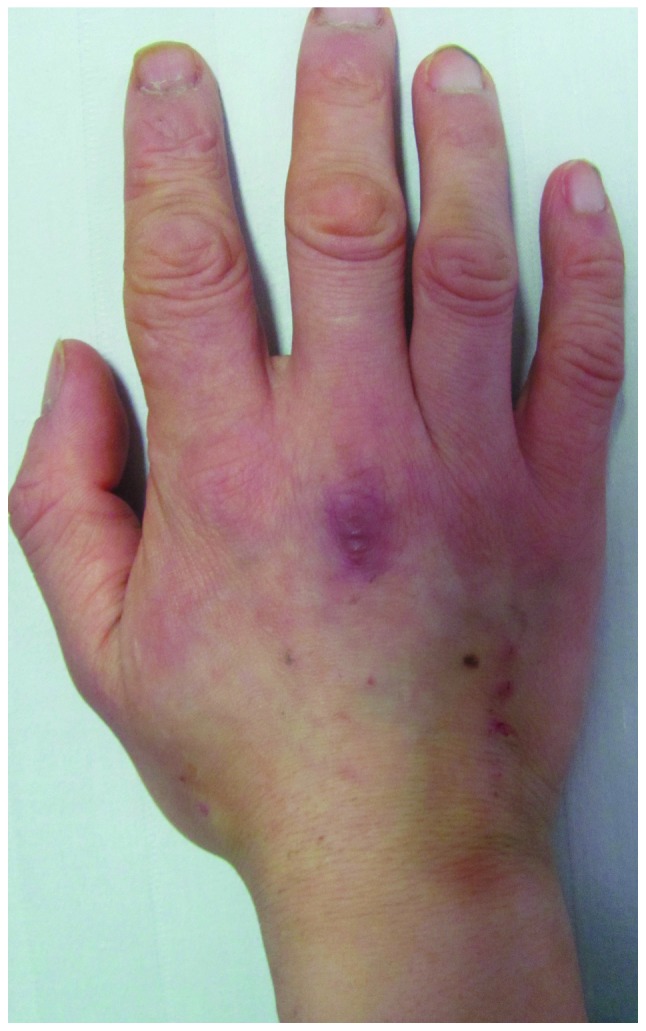
Erythematous nodule resulting from cutaneous vasculitis.

**Figure 2:**
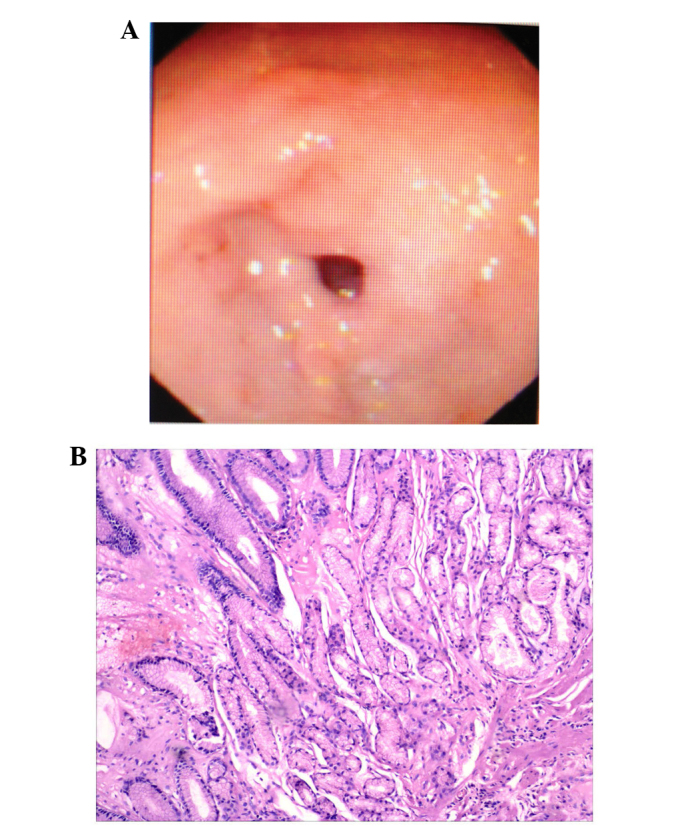
(A) Upper gastrointestinal endoscopic findings. Small ulcers with edema in the surrounding mucosa were observed in the lesser curvature of the antrum (magnification, ×1.6). (B) Histopathological findings of an endoscopy biopsy specimen (Hematoxylin and eosin staining). Mucosal inflammation of the gastric antrum was mild and exhibited no specific features (magnification, ×100).

In November 2012, a 65-year-old male was admitted to the First Affiliated Hospital of Chongqing Medical University (Chongqing, China) with dizziness that had persisted for one month and described the occurrence of melena for two weeks. The physical examination was notable for pallor and rashes, and the patient had developed erythematous and maculopapular skin lesions on the legs and hands (Fig. 1). The patient refused to undergo a skin biopsy. Maculopapular rashes appeared around the injection site minutes after intravenous injection. Blood tests were performed and the results were as follows: Hemoglobin level, 6.4 g/dl; hematocrit, 19.10%; platelet count, 58×10^9^/l; and total leukocyte count, 4.55×10^9^/l, with 8% blast cells and 6% mature basophils. The fecal occult blood test was positive, the level of ferritin was 1,138 ng/ml and the levels of vitamin B12 and folic acid were normal. Serum autoantibodies, including the anti-neutrophil cytoplasmic antibodies (ANCA), myeloperoxidase (MPO)-ANCA and proteinase 3-ANCA, were negative. A gastroscopy demonstrated a gastric antral ulcer (stage, A1), according to Sakita’s endoscopic staging system (2) and the endoscopy biopsy revealed mild mucosal inflammation and no specific features. (Fig. 2A and B).

**Figure 3:**
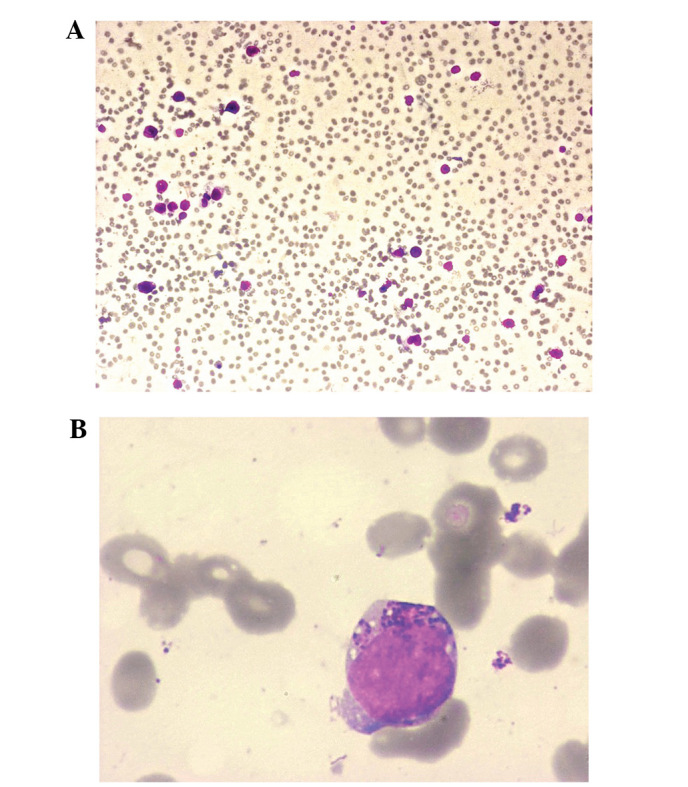
(A) Bone marrow aspirate demonstrating acute basophilic leukemia blast cells (magnification, ×100). (B) Certain blast cells demonstrated basophilic granulations. (Stain, Wright-Giemsa; magnification, ×1000).

**Figure 4:**
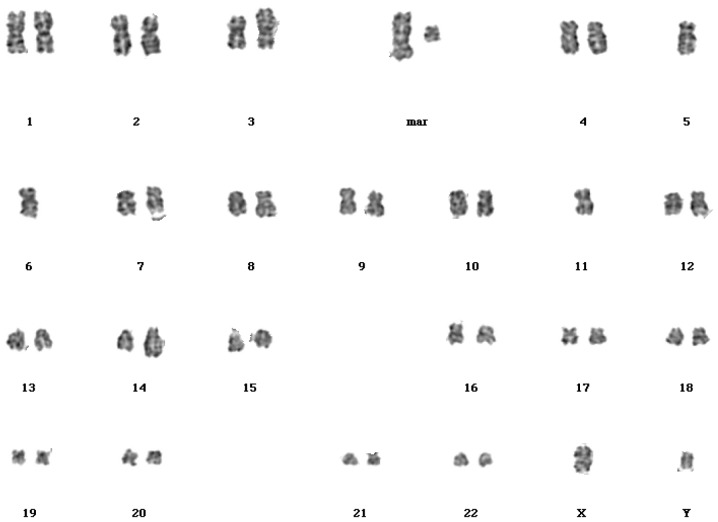
Cytogenetic studies using GTG banding revealed an abnormal karyotype, 44 XY,add(3)(p26),-5,-11,add(14)(q32),+mar1,+mar2,inc[18]/46,XY[2].

**Figure 5:**
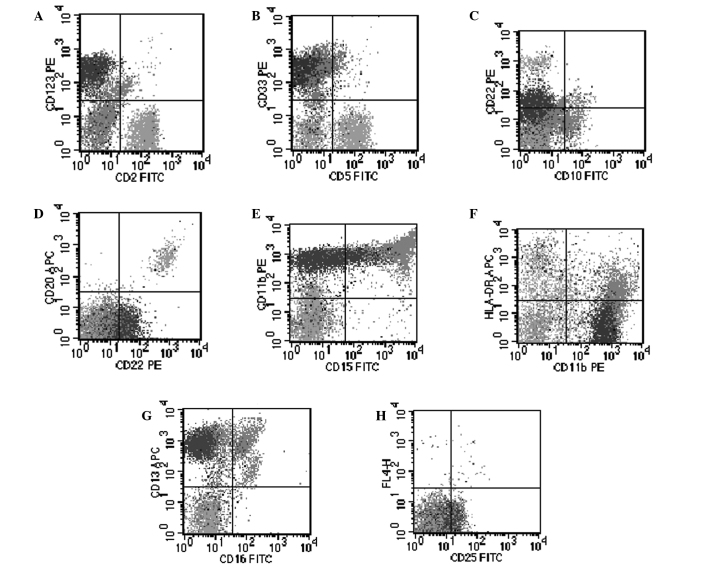
Dot plots showing immunoreactivity pattern of blast cells. Basophilic cells account for 38% of the total nucleated cells. Blast cells showing positivity for (A) CD123, (B) CD33, (C and D) CD22, (E and F) CD11b, (G) CD13 and (H) CD25. CD, cluster of differentiation.

The results of a bone marrow aspirate (stain, Wright-Giemsa) were as follows: Blast cells, 13%; atypical immature basophils, 14%; mature basophils, 8%; normal mature neutrophils, 11%; and erythroid cells, 13%. The blast cells contained basophilic granules, and the vacuoles in the cytoplasm and nuclei were oval and bilobed and contained nucleoli (Fig. 3A and B). The blast cells were positive for peroxidase and periodic acid-Schiff staining (PAS) and negative for α-naphthyl acetate esterase. The blast cells exhibited metachromasia as a result of staining with toluidine blue. Six days later, the bone marrow aspirate with Wright-Giemsa stain was conducted again, and showed 13% blast cells, 16% atypical immature basophils and 8% mature basophils, with 9% normal mature neutrophils. Cytogenetic studies using GTG banding revealed an abnormal karyotype, 44 XY,add(3)(p26),-5,-11,add(14)(q32),+mar1, +mar2,inc[18]/46,XY[2] (Fig. 4). Flow cytometric analysis of the blast cells was positive for cluster of differentiation (CD)11b, CD13, CD22, CD25, CD33 and CD123 (Fig. 5). BCR-ABL fusion was identified to be negative by reverse transcription-polymerase chain reaction analysis and no c-kit D816V mutation was detected.

Following the above-mentioned assessments, the diagnosis of ABL was determined. Induction therapy with daunorubicin (45 mg/m2 daily) intravenously for three days and cytarabine (100 mg/m^2^ daily) intravenously via continuous infusion for seven days was initiated at the First Affiliated Hospital of Chongqing Medical University (Chongqing, China) on day 13. The hemoglobin level was 5.7 g/dl; the hematocrit was 17.10%; the platelet count was 40×10^9^/l; and the total leukocyte count was 4.41×10^9^/l, with 4% blast cells and 4% basophils. Loratadine, cimetidine and steroids were introduced into the treatment regimen due to concerns regarding the patient’s increased histamine levels and the rashes and melena subsequently disappeared. The patient’s bone marrow examination on day 21 was hypocellular with 1% blast cells, 6% atypical immature basophils and 19% mature basophils, with 24% normal mature neutrophils. The hemoglobin level was 7.4 g/dl; the hematocrit was 22.80%; the platelet count was 31×10^9^/l; and the total leukocyte count was 2.95×10^9^/l, with no blast cells and 12.2% mature basophils. The rashes reappeared, therefore, reinduction therapy was initiated with idarubicin (8 mg/m^2^ daily) intravenously for three days and cytarabine (100 mg/m^2^ daily) intravenously via continuous infusion for seven days. Three weeks later, the bone marrow aspirate (stain, Wright-Giemsa) showed 3% blast cells, 8% atypical immature basophils and no mature basophils, with 58% normal mature neutrophils. The hemoglobin level was 6.9 g/dl; the hematocrit was 21.00%; the platelet count was 34×10^9^/l; and the total leukocyte count was 3.71×10^9^/l, with no blast cells and 0.8% mature basophils. The patient was discharged from hospital. However, one month later, the patient contracted a severe pneumonia infection and succumbed as a result of a multiorgan system failure without evidence of relapse.

## Discussion

Diagnosing *de novo* ABL can be challenging (3). It was hypothesized that the patient described in the present case was diagnosed with *de novo* ABL due to the negative results that were obtained from assessing the BCR-ABL transcripts and because there were no previous events that were indicative of a blood count abnormality. *De novo* ABL was first described by Wick *et al* (3) and similar cases have subsequently been reported by others (4,5). In the WHO classification of myeloid malignancies (2008 revision) (1), ABL has been integrated as a distinct entity and defined as an acute myeloid leukemia (AML) in which the primary differentiation is to basophils. A peripheral blood smear should reveal basophilia and the bone marrow may demonstrate blast cells, often in addition to immature basophilic granules; mature basophils may or may not be observed. The blast cells may stain positively for PAS, MPO and toluidine blue (6). Furthermore, immunophenotyping may identify a basophilic lineage (4). ABL blast cells exhibit a myeloid phenotype and the following antigens may be expressed: CD9, CD45, CD13, CD17, CD33, CD123, CD11b, and CD25. In addition, ABL is not associated with any specific chromosomal abnormality; however, cytogenetic studies and molecular analysis are required in all such cases to rule out blast crisis of chronic myeloid leukemia (CML) as most cases of ABL develop secondary to CML (7–9).

Differentiation of basophils from mast cells is not complicated, however, may be problematic when evaluating leukemic populations and, therefore, mast cell leukemia should be considered in the differential diagnosis of ABL. While mature basophils are positive for CD25 and negative for CD117, mast cells are positive for CD117 and negative for CD25 (10). In complex cases, electron microscopy may aid with discriminating basophils from mast cells (6). Although CD25 is characteristic, it is not specific to basophil lineage of blast cells, as CD25 positivity has been observed in blast cells of CML, and a small number of cases of myeloblastic and BCR-ABL-positive lymphoblastic leukemia (11). Furthermore, the c-kit D816V mutation is detected in the majority of adult cases of systemic mastocytosis (SM), including mast cell leukemia, which is one of the minor criteria for a diagnosis of SM, according to the 2008 WHO classification of myeloproliferative neoplasms (12). In the present case, the characteristic cytomorphological features, the myeloid immunophenotype of the blast cells with distinct maturation patterns and positivity for CD25 and CD123, along with the absence of the Philadelphia chromosome and the c-kit D816V mutation favored a diagnosis of ABL and ruled out basophophilic blast crisis of CML, acute lymphoblastic leukemia, non-basophil lineage AML, AML with t(6;9)(p23;q34) (13) and mast cell leukemia.

The maculopapular skin lesions on the patient’s legs and hands may have been due to vasculitis, although no skin biopsy was performed. The common hematologic malignancies that are associated with secondary vasculitis include myelodysplastic syndrome, lymphomas and hairy cell leukemia. In the majority of cases they are ANCA negative (14). In individuals presenting with ABL, the high blood levels of histamine may elicit cutaneous signs, including pruritus, edema, maculopapular rashes, areas of hyperpigmentation and/or digestive signs, such as nausea, vomiting, diarrhea or ulcers. The patient’s melena, gastric antral ulcer and cutaneous vasculitis may have resulted from an anaphylactic reaction.

The treatment combination of daunorubicin/idarubicin and cytarabine was selected with the addition of H1- and H2-receptor antagonists and steroids introduced later in the treatment regimen. In ABL, potential serious complications associated with a massive release of histamines from the degranulating basophilic cells must be addressed. These effects may include shock or anaphylaxis, as well as gastric acid hypersecretion, peptic ulcerations or gastrointestinal bleeding. Although the prognosis was poor, the patient achieved a certain level of clinical improvement on initiation of the treatment.

In conclusion, as a rare subtype of AML, ABL is diagnosed with difficulty and associated with unique therapeutic complications, including anaphylaxis, and gastroduodenal and cutaneous involvement. Recognition of the occurrence of ABL enables the appropriate prophylactic measures to be taken, including the administration of H1- and H2-receptor blockers, proton pump inhibitors and steroids, which may aid with minimizing the treatment-associated complications.
